# How does removing menthol tobacco product displays at point-of-sale affect adolescents’ cigarette smoking intentions? The mediating effects of social norms

**DOI:** 10.1007/s10865-025-00551-4

**Published:** 2025-02-20

**Authors:** Jody Chin Sing Wong, Claude Messan Setodji, Michael S. Dunbar, Steven Martino, Grace van Valkenburg, Desmond Jenson, William G. Shadel

**Affiliations:** 1 Behavioral and Policy Sciences, RAND Corporation, 4570 Fifth Ave #600, Pittsburgh, PA USA; 2 Public Health Law Center, Mitchell Hamline School of Law, Saint Paul, MN 55105 USA

**Keywords:** Cigarettes smoking intention, Injunctive norms, Mediation, Social norms, Tobacco advertising

## Abstract

This study investigates the psychological mechanisms through which a removal of mentholated tobacco products from retail stores affects future smoking intentions among youth. Descriptive norms and injunctive norms were examined as candidate mediators. The study was conducted in the RAND StoreLab (RSL), a life-sized replica of a convenience store developed to evaluate how changing point-of-sale (POS) tobacco advertising influences tobacco use outcomes during simulated shopping experiences. Participants were assigned to shop randomly in the RSL under one of three experimental conditions that were (1) status quo condition in which all tobacco-, sweet-, and menthol-flavors were displayed; (2) tobacco/menthol condition in which only tobacco- and menthol-flavored tobacco products were displayed (sweet characterizing flavors other than tobacco or menthol/mint were removed from the display, effectively “banned”); and (3) tobacco-only condition in which only tobacco-flavored products were displayed (all sweet- and menthol-flavored products were removed). Results revealed that injunctive norms mediated the relationship between the removal of menthol cigarettes from the POS setting and increased intentions to smoke menthol-flavored cigarettes, whereas descriptive norms were not a significant mediator. These findings suggest that targeting injunctive smoking norms in public health communications may be a promising strategy to mitigate potential unintended consequences of a menthol ban on future smoking intentions for youth.

## Introduction

The U.S. is in the process of considering regulations that could formally ban the sale of menthol cigarettes. Although the enactment of this policy is currently on an indefinite hold, such a change would likely have significant effects on consumers’ thoughts and feelings about cigarettes and their perceptions of the tobacco retail marketplace. Understanding more about the implications of removing menthol tobacco products from the point-of-sale, particularly on young people, is crucial for anticipating the outcomes of such regulatory actions. Young people, particularly those between the ages of 11 to 24 (adolescence through early young adulthood), are very frequent users of menthol cigarettes (Giovino et al., 2015) and an important population to study in this regard.

Findings from studies of adult smokers in countries outside of the U.S. where menthol bans have been implemented offer some insight in this regard but are mixed: bans are associated with quitting among menthol smokers but are also associated with menthol smokers changing to nonmenthol cigarettes and/or continuing to use menthol cigarettes (Chaiton et al., 2020; Liber et al., 2022). Almost no research has been conducted to understand how a menthol ban would affect young people in the U.S., a population known to favor menthol cigarettes (Mantey et al., 2021) and to be more likely to persist with smoking if they start by first using menthol cigarettes (Cwalina et al., 2020); Leas et al., 2022). One of the only experimental studies that evaluated the effects of menthol bans in young people found that removing menthol-flavored tobacco products from the retail setting led to stronger intentions to use menthol-flavored cigarettes, an effect that was more pronounced among individuals with a higher baseline risk of tobacco use (Shadel et al., 2023). This counterintuitive finding clearly foreshadows a highly undesirable consequence of any regulatory policy that bans the sale of menthol cigarettes. For example, young people could seek menthol cigarettes on the black market (e.g., Wackowski et al., 2018) or gravitate toward newer brand cigarettes that mimic the taste and sensations of menthol (Meza et al., in press). As such, it is critical to throw additional light on the unexpected effect in Shadel et al., (2023) to understand more about it and to uncover the psychological mechanisms that contributed to it. Indeed, identifying the psychological mechanisms that mediate youths’ responses to menthol cigarette bans could help to identify potential targets for health behavior interventions designed to help youth manage menthol use intentions that rise in the face of a ban.

The purpose of this study was to evaluate two well-studied studied psychological constructs, perceived *descriptive norms* and *injunctive norms*, as candidate mediators of the effects observed in Shadel et al. (2023). Social norms theory (Berkowitz, 2004) assigns powerful regulatory roles to descriptive norms and injunctive norms. These constructs are linked to smoking uptake among young people (East et al., 2021) and are increasingly targeted by smoking prevention interventions (e.g., Campbell et al., 2008; Chou et al., 2006). *Descriptive norms* are beliefs about the perceived prevalence of a behavior in a specific reference population (e.g., family or peers). Perceived descriptive norms are associated with cigarette smoking among young people: the more of their same-aged peers that they believe smoke, the more likely they are to smoke (Brown et al., 2010). Moreover, perceived descriptive norms mediate the relationship between exposure to point-of-sale tobacco displays and adolescents’ cigarette smoking intentions (Setodji et al., 2018): exposure leads to adolescents believing that more of their peers smoke, and those increases in their estimates of peer smoking contribute to increases in their intentions to smoke. *Injunctive norms* refer to beliefs about the extent to which a given behavior is acceptable or unacceptable among an individual’s social network. Greater perceived acceptability of smoking is associated with stronger smoking intentions (e.g., Zaleski & Aloise-Young, 2013) and smoking behavior (e.g., Etcheverry et al., 2008), and injunctive norms are important for understanding the effects of point-of-sale tobacco counter-marketing interventions on young people’s smoking intentions (Dunbar et al., 2019). Given the established role of descriptive and injunctive norms in youth smoking, we hypothesized that both descriptive and injunctive norms would mediate the relationship between their exposure to a retail environment that banned menthol tobacco products and young people’s menthol cigarette use intentions.

## Methods

### Overview

Full details of the main study experiment can be found in Shadel et al. (2023). A sample of young people (N = 230), ages 11–20, was recruited from the community and randomly assigned to shop in the RAND Storelab (RSL) under either one of three between-subjects conditions that varied the composition of tobacco products displayed. A (1) *status quo condition* in which all tobacco-, sweet-, and menthol-flavors were displayed **(TSM-F)**; (2) *tobacco/menthol condition* in which only tobacco- and menthol-flavored tobacco products **(TM-F)** were displayed (i.e., sweet characterizing flavors other than tobacco or menthol/mint were removed from the display, effectively “banning” them for sale in the RSL), and (3) *tobacco-only condition* in which only tobacco-flavored products **(T-F)** were displayed (i.e., all sweet- and menthol-flavored products were banned). The characteristics of the tobacco powerwall, such as its size and position, the count of tobacco packages shown, and the placement and details of the price tags and posters, remained consistent across all conditions. After shopping in the RSL, participants completed measures of descriptive norms, injunctive norms, and future smoking intentions (described below). Those who completed the study received a $40 gift card as compensation. All research procedures were approved by the institutional review board (IRB) at the authors’ institution.

## Sample

Participants were recruited from Pittsburgh, PA, through media advertisements focused on habits related to shopping at convenience stores. No smoking or tobacco were mentioned in any advertising materials. Prospective participants underwent a telephone screening to determine their eligibility. To qualify, individuals needed to be between the ages of 11 and 20, have no medical or psychological issues that would impede study involvement, and must not have participated in any prior study conducted in the Storelab (see for more, Shadel et al., 2012, 2016, 2019a, 2019b).

## Experimental setting

The RAND Storelab is a 1500 ft^2^ facility within an office building in Pittsburgh, PA, designed to simulate a realistic convenience store environment. This setting allowed us to observe participants’ interactions with point-of-sale displays without the influence of external variables present in actual retail environments. The store houses a diverse inventory of over 650 items (e.g., dairy products, snacks, beverages, tobacco), where posters are placed throughout the RSL for products such as soda, food, and candy. Tobacco posters are located on the storefront and on the tobacco powerwall. The tobacco powerwall display sits behind the checkout counter, mirroring real-world point-of-sale displays that participants might encounter outside the study.

## Study procedure

At the beginning of the study, all participants (and parents, if minors) were presented with the informed consent and a set of instructions regarding the procedure of the experiment (the study used an authorized deception to help mask the true purpose of the research). They first completed a baseline questionnaire that assessed information such as demographics, and lifetime tobacco use. In addition, filter items (e.g., soda and salty snacks rather than tobacco products) were administered to help to further mask the true purpose of the study.

Next, participants were randomly assigned to experimental conditions and individually shopped in the Storelab. They were given a $10 gift card for the RSL and invited to shop freely, selecting any items they desired for an unlimited amount of time. They were required to buy at least one product and check out as they would in a typical convenience store. A confederate, who was not part of the consent or survey process, played the role of the cashier. This cashier processed the items for payment, scanned the gift card, and packaged the purchases. While no attempts were made by participants to buy tobacco products, any such efforts would have been denied by the cashier, who would have asked for identification to verify age, and later refused the sale, as all participants were under 21 years old.

Upon leaving the Storelab, participants completed the assessments of the mediators and dependent variables along with structurally similar filler items (again to help mask the study purpose). They then participated in a detailed debriefing process, during which they were asked to speculate on the study’s objectives through an open-ended question. Participants were informed that they were not allowed to keep the items they purchased. Lastly, they watched a brief video on tobacco media literacy and were provided with printed materials on smoking prevention. The video and printed materials were provided to educate participants on the risks associated with tobacco use. The effects of the giving of this information were not a subject of our investigation. At the conclusion of the session, they were compensated with a $40 gift card. At the conclusion of the session, they were compensated with a $40 gift card.

## Measures

***Pre-RSL*** ***shopping.***

*Demographics***.** Participants self-reported information related to their age, education, gender, race, and ethnicity.

*Lifetime Tobacco Use*. A single item evaluated participants’ lifetime/ever use of each of four tobacco product classes that were cigarettes, ENDS, LCCs, smokeless tobacco: “Have you ever used/smoke [product] in your life?” They selected one of two response that was either “yes” or “no”. (Patrick et al., 2022). Participants that responded “no” to all four products were coded as never users; anyone that responded “yes” to any of the four products was coded as a tobacco user.

*Smoking Intentions***.** A 3-item scale adapted from prior research (Choi et al., 2001; Pierce et al., 1996; Setodji et al., 2024) evaluated participants’ smoking intentions for *menthol-flavored* cigarettes: “Do you think you will try a cigarette flavored with menthol or mint anytime soon?”, “Do you think you will smoke a cigarette flavored with menthol or mint anytime in the next year?”; and “If one of your best friends offered you a cigarette flavored with menthol or mint, would you smoke it?”. Each item was rated on a 1 = definitely not to 10 = definitely yes scale and responses to the three items were summed. Higher scores indicated greater risk of future smoking (αs > 0.91). Because the distribution of scores on this measure was skewed (i.e., over 77% of participants had the lowest score, i.e., a ‘3’), scores were dichotomized: those who scored a ‘3’ were recoded as ‘0’ (no risk) and any scores greater than ‘3’ were coded as ‘1’ (at risk). This scoring convention is commonly used for this assessment (Choi et al., 2001; Shadel et al., 2023).

***Post-RSL*** ***shopping.***

### Mediators

*Descriptive Norms***.** A single item evaluated descriptive norms for *menthol-flavored* cigarettes: “What percentage of kids in your grade smoke cigarettes with menthol or mint flavors?” Based on their estimation, they specified the percentage (Unger et al., 2001).

*Injunctive Norms***.** A single item rated on a 4-point Likert scale from 1 = strongly disagree to 4 = strongly agree assessed injunctive norms for *menthol-flavored* cigarettes: “According to my friends, it is very important for me to not smoke cigarettes with menthol or mint flavors.” (Primack et al., 2007). Responses were reversed scored so that higher scores indicated greater perceived social approval of menthol cigarette smoking.

### Dependent measure

*Smoking Intentions***.** The same cigarette smoking intentions items, given at the pre-RSL shopping point, was administered during the post shopping period as the dependent variable. As with the baseline variable, intentions were coded so that scores of ‘3’ were recoded as ‘0’ (no risk) and scores greater than ‘3’ were coded as ‘1’ (at risk).

## Analytic plan

A series of mediation analyses were conducted (Imai et al., 2010; see also, Baron & Kenny, 1986; Setodji et al., 2018) to investigate whether descriptive norms and/or injunctive norms mediated the relationship between study condition and the risk of smoking menthol cigarettes. For each of the two hypothesized mediator variables (M), we used regression models to test whether the mediators were affected by the experimental conditions (tobacco/menthol **TM-F** or tobacco-only **T-F** condition compared to the status quo **TSM-F**) and also whether each influenced the post-shopping intentions to smoke menthol cigarettes (Y) in a way that explained in whole or in part the effect of study condition. First, we regressed the outcome Y on the experimental conditions and each mediator, controlling for covariates summarized by X (age, education, gender, race, and baseline smoking intentions):

1 $$g\left( Y \right) = \mu + \lambda_{1} {\text{Condition}}_{TM - F} + \lambda_{2} {\text{Condition}}_{T - F} + \beta {\text{Mediator}} + \gamma_{1} X + \varepsilon_{y}$$

where g() is the non-linear logistic regression transformation used because of the binary outcome. Then, each mediator M was regressed on study condition, again controlling for the covariates X:

2 $${\text{Mediator}} = \nu +_{1} {\text{Condition}}_{TM - F} +_{2} {\text{Condition}}_{T - F} + \gamma_{2} X + \varepsilon_{m}$$

The common mediation approach (Baron & Kenny, 1986) is for bimodal experimental conditions but for this multicategorical experimental condition, the approach proposed by Hayes and Preacher (2014) was used and all estimates will be referred to in “relative to the status quo” terms. Also, because the outcome (intentions) is binary, estimation of the mediation parameters is achieved using the counterfactual framework of causal mediation effects proposed by Imai et al. (2010). Statistical analyses were conducted using the R package (version 4.4.1) for Causal Mediation Analysis (Tingley et al., 2014) along with a nonparametric bootstrap procedure with 10,000 replications to determine statistical significance.

## Results

Randomization balanced key characteristics across conditions. The average age of participants in 16.56 (*SD* = 3.11) with 65.2% of them being female and most of them having graduated from high school (54.9%). Most participants (62.0%) identified as White, 16.54% identified as Asian, 12.03% identified as Black, and 6% identified as Hispanic/Latino. About 16% of the sample reported any tobacco use.

## Assessing the mediating role of injunctive norms

Table 1 presents these results. Logistic regression analysis revealed that being in the tobacco-only condition (T-F) led to a significant increase in participants’ menthol smoking intentions relative to the status-quo while the being in the tobacco/menthol condition (TM-F) was not significantly different compared to the status-quo (Table 1). In addition, being in the tobacco only (T-F) condition led to an increase in injunctive norms when compared to the status quo (i.e., being in the condition where menthol products were removed led to stronger perceptions of approval of menthol cigarette smoking). Effects were similar in direction, but stronger, when restricting the sample to participants that had some past use of tobacco.

**Table 1:** Results of logistic regression outcome models and linear mediation models testing injunctive norms as mediator between exposure to point-of-sale advertising and menthol cigarette smoking intention

Sample	Variables	Outcome model (logistic)			Mediator model (linear)		
*Full*	Predictor	Estimate	SE	*p* value	Estimate	SE	*p* value
	Mediator: Injunctive norms	0.64	0.30	**0.033**			
	Tobacco/menthol condition **TM-F**	0.70	0.61	0.255	− 0.07	0.13	0.595
	Tobacco-only condition **T-F**	1.47	0.65	**0.024**	0.36	0.13	**0.008**
	Covariate: Lifetime tobacco use	1.41	0.60	**0.018**	0.22	0.14	0.112
	Age	0.42	0.20	**0.041**	0.06	0.04	0.137
	Education	− 1.69	1.12	0.131	− 0.14	0.24	0.561
	White	0.37	0.51	0.469	0.01	0.11	0.941
	Female	− 0.43	0.54	0.426	− 0.09	0.12	0.447
	Baseline smoking intention	3.64	0.58	**0.000**	0.16	0.13	0.214
*Tobacco-Users Only*							
	Mediator: Injunctive norms	1.67	0.66	**0.011**			
	Tobacco/menthol condition** TM-F**	2.35	1.04	**0.024**	0.10	0.20	0.629
	Tobacco-only condition** T-F**	1.87	1.08	0.083	0.47	0.22	**0.036**
	Age	0.63	0.37	0.090	0.00	0.08	0.968
	Education	0.76	1.62	0.638	− 0.18	0.40	0.656
	White	0.83	0.76	0.276	0.24	0.18	0.192
	Female	1.49	1.01	0.142	− 0.41	0.20	**0.039**
	Baseline smoking intention	4.23	1.00	**0.000**	0.56	0.17	**0.002**

Sig. results are **bolded**

Table 3 (top two rows) presents the results of the decomposition analysis. The total tobacco-only (T-F) effect increased menthol cigarette smoking intentions, relative to the status quo, by 13.6%, 95% CI [3.3%, 23.7%], *p* = 0.010 (in the full sample), and a significant portion of that effect (1.8%, 95% CI [0.0%, 4.4%], *p* = 0.015 or 12.5% of the total effect) was mediated by increases in injunctive norms. The effect among tobacco users was stronger: While the total tobacco-only (T-F) effect in this subgroup increased menthol cigarette smoking intentions, relative to the status quo, by 23.1%, 95% CI [2.3%, 40.1%], the level of the effect mediated through the injunctive norms was 7.0%, 95% CI [0.5%, 15.9%], or 16.2% of the total effect.

## Assessing the mediating role of descriptive norms

**Fig. 1:**
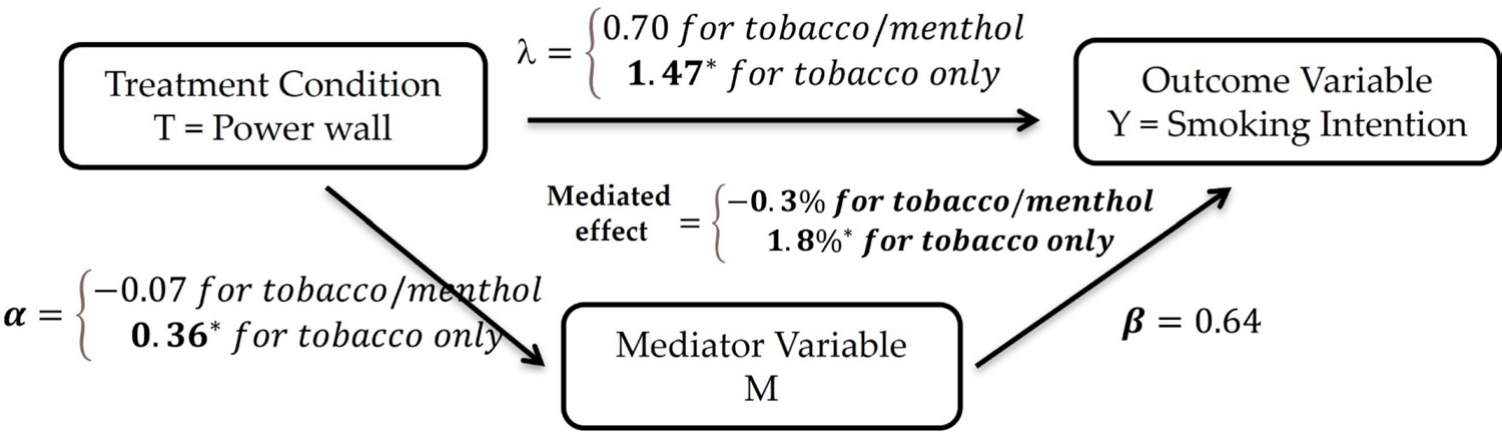
The effect decomposition for the full sample with injunctive norm as mediator

Table 2 presents results of our analysis of for the mediating role of descriptive norms. Exposure to the tobacco-only condition (T-F) led to a significant increase in menthol smoking intentions. However, the impact of study condition on menthol cigarette smoking intentions while controlling for descriptive norms was not significant. The decomposition analysis (bottom rows, Table 3) was consistent with these results. The total increase in menthol smoking intentions of 13.4%, (95% CI [3.4%, 23.4%], *p* = 0.010) attributable to the tobacco-only condition (T-F) compared to the status quo yielded a non-significant mediated effect of size -0.2%, (95% CI [-1.3%, 0.6%], *p* = 0.73). Even when restricted to the tobacco users, the mediated effect was only of size 0.5% (95% CI [-4.3%, 6.0%], *p* = 0.81). (Fig. 1)

**Table 2:** Results of logistic regression outcome models and linear mediation models testing descriptive norms as mediator between exposure to point-of-sale advertising and menthol cigarette smoking intention

Sample	Variables	Outcome model (logistic)			Mediator model (linear)		
*Full*	Predictor	Estimate	SE	*p* value	Estimate	SE	*p* value
	Mediator: Descriptive norms	0.02	0.02	0.256			
	Tobacco/menthol condition** TM-F**	0.69	0.59	0.247	− 0.33	2.18	0.881
	Tobacco-only condition** T-F**	1.70	0.64	**0.008**	− 1.25	2.21	0.574
	Covariate: Lifetime tobacco use	1.64	0.58	**0.005**	1.63	2.29	0.476
	Age	0.46	0.21	**0.027**	1.64	0.65	**0.013**
	Education	− 2.10	1.17	0.073	4.78	3.97	0.230
	White	0.41	0.51	0.424	− 4.03	1.87	**0.033**
	Female	− 0.65	0.52	0.217	0.29	1.92	0.879
	Baseline smoking intention	3.63	0.56	**0.000**	1.59	2.15	0.461
*Tobacco-Users Only*							
	Mediator: Descriptive norms	0.04	0.03	0.137			
	Tobacco/menthol condition** TM-F**	2.17	1.02	**0.033**	0.42	4.28	0.923
	Tobacco-only condition** T-F**	2.20	1.02	**0.030**	1.07	4.79	0.824
	Age	0.55	0.41	0.176	2.17	1.73	0.215
	Education	− 0.23	1.92	0.903	5.90	8.66	0.498
	White	1.11	0.77	0.150	− 7.06	3.90	0.075
	Female	0.09	0.79	0.907	7.08	4.18	0.095
	Baseline smoking intention	4.52	1.01	**0.000**	3.09	3.79	0.418

Sig. results are **bolded**

**Table 3:** Causal effect decomposition of the relationship between experimental conditions and menthol cigarette smoking intention

	Tobacco/menthol TM-F condition Effect Decomposition relative to Status Quo				Tobacco-only T-F condition Effect Decomposition relative to Status Quo			
Effect type	Effect	Low CI	Up CI	*p*	Effect	Low CI	Up CI	*p*
Full Sample Hypothesized mediator: Injunctive norms								
Total effect	5.5%	− 4.8%	16.3%	0.25	13.6%	3.3%	23.7%	0.01
Average causal mediated Effect	− 0.3%	− 2.0%	1.2%	0.64	1.8%	0.0%	4.4%	0.05
Average causal direct effect	5.8%	− 4.2%	16.5%	0.22	11.8%	1.3%	21.8%	0.02
Tobacco-Users Only Sample Hypothesized mediator: Injunctive norms								
Total effect	21.5%	3.0%	39.0%	0.03	23.1%	2.3%	40.1%	0.03
Average causal mediated Effect	1.5%	− 4.3%	8.2%	0.64	7.0%	0.5%	15.9%	0.02
Average causal direct effect	20.0%	1.9%	35.8%	0.03	16.2%	− 3.5%	32.7%	0.08
Full Sample Hypothesized mediator: Descriptive norms								
Total effect	5.6%	− 4.4%	16.0%	0.21	13.4%	3.4%	23.4%	0.01
Average causal mediated effect	0.0%	− 1.1%	0.8%	0.96	− 0.2%	− 1.3%	0.6%	0.73
Average Causal Direct Effect	5.7%	− 4.2%	15.9%	0.21	13.6%	3.7%	23.1%	0.01
Tobacco-Users Only Sample Hypothesized mediator: Descriptive norms								
Total Effect	20.9%	1.3%	38.6%	0.04	22.2%	2.2%	38.6%	0.03
Average Causal Mediated Effect	0.3%	− 4.2%	5.2%	0.93	0.5%	− 4.3%	6.0%	0.81
Average Causal Direct Effect	20.6%	1.4%	38.1%	0.04	21.7%	2.0%	38.1%	0.04

## Discussion

A previous study (Shadel et al., 2023) found that removing menthol flavored tobacco products from an experimental convenience store led to increases in intentions to smoke menthol cigarettes in young people. However, the potential mechanisms underlying this counterintuitive effect were previously unknown. The present study provided crucial insight into this observed relationship, finding that injunctive norms were a significant mediator of this effect: removing menthol products led young people to believe that their friends were more approving of menthol smoking, which in turn, led to increases in their intentions to smoke menthol cigarettes. The link between greater perceived peer approval of smoking behavior and increased likelihood of smoking is well established (East et al., 2021), so our finding a positive relationship between injunctive norms about menthol cigarette smoking and intentions to use menthol cigarettes is not surprising. The finding that removing menthol cigarettes from the RSL led to greater perceived peer approval of menthol cigarette smoking may appear counterintuitive. However, consider that injunctive norms are largely prescriptions for what people should and should not do, and could be construed as restricting their freedom of choice, leading to psychological reactance (Steindl et al., 2015). Given that menthol cigarettes are strongly preferred, and their use is highly prevalent among young people (Mantey et al., 2021), removal of menthol cigarettes from the RSL may have conveyed the message that these products are not to be used. This, in turn, may have led participants to believe that their freedom of choice regarding menthol cigarettes was being threatened, and led to psychological reactance which was then reflected in greater perceived acceptance of menthol cigarettes by their peers. We did not measure psychological reactance directly, so this interpretation is speculative. However, findings are consistent with other, seemingly counterintuitive findings in the literature. For example, Nan and Zhao (2016) found that exposure to anti-smoking messages led to increased perceived peer approval of smoking, which then led to greater smoking intentions.

Descriptive norms did not act as a mediator. Extent research show that descriptive norms, which are grounded in more observable behaviors, are often inaccurately perceived by young individuals and may require more intensive and targeted interventions (e.g., normative feedback) for meaningful change, as brief exposures may not suffice. Moreover, young people tend to overestimate the prevalence of behaviors such as alcohol consumption and smoking, leading to ceiling effects that limit intervention effectiveness. While brief interventions providing accurate information can reduce these behaviors by diminishing social pressure, more comprehensive strategies such as educational campaigns, social norms marketing, and peer-led discussions are essential. Correcting these misperceptions is crucial, but it is challenging due to the frequent overestimation of peer behaviors, as highlighted by existing literature on adolescents’ smoking norms.

Overall, then, these findings imply that health education and prevention programs aimed at young people should, in the face of a menthol cigarette ban, focus on correcting their perceptions of peer acceptance of menthol cigarette smoking and perhaps, working to mitigate the effects of psychological reactance. For example, emphasizing undesirability of menthol smoking norms among peers and showcasing admired smoke-free role models could reinforce positive behaviors (Liu & Yang, 2024). In addition, openly discussing the reasons behind menthol bans and the benefits they could bring may help to mitigate the ‘forbidden fruit’ appeal of smoking, to protect against psychological reactance. For instance, one novel and highly innovative way could be employing narrative messages that could provoke character identification with the protagonists and reactance reduction (Green & Brook, 2000). Indeed, clinicians and public health professionals might play a crucial role in addressing the implications of menthol cigarette bans. Although the specific communication strategies that clinicians could use would need to be developed and tested to ensure efficacy, clinicians could, for example, emphasize the health risks associated with menthol smoking, while at the same time addressing any perceived loss of freedom prompted by a menthol ban. Moreover, integrating discussions about the broader benefits of tobacco control measures, such as improved community health outcomes and reduced healthcare costs, could further support public health goals.

As with all research, this study has its limitations. We first note the contrived experimental setting of the RSL. Although it has its merits in that it is closely modeled after a real convenience store, it is still an artificial one. Concomitantly, participants were exposed to the respective point-of-sale environment only once (as opposed to repeated exposures). Thus, the RSL would not necessarily provide a good environment to model the entire process of how the presence or absence of tobacco products influences youth smoking. Second, the use of single-item measures for descriptive norms and injunctive norms could have affected our results; longer measures are available (Primack et al., 2007). Third, our sample were mostly non-Hispanic Whites and based in Pittsburgh, potentially limiting the generalizability of our findings.

In summary, this study sheds light on the complex dynamics between the removal of menthol cigarettes and young people’s smoking intentions. Our findings highlight the role of injunctive norms as a mediator, suggesting that perceived peer approval can influence smoking behaviours even in the context of regulatory measures like menthol bans. The potential for psychological reactance to influence young people’s perceptions and behaviours presents both a challenge and an opportunity for clinicians and public health professionals. By understanding the psychological mechanisms highlighted in this study, stakeholders can design more effective strategies to counteract unintended consequences and promote smoke-free behaviours. This research contributes valuable insights to the ongoing discourse on tobacco control and youth smoking prevention.
